# Open-source software for data science

**DOI:** 10.1016/j.patter.2025.101324

**Published:** 2025-07-11

**Authors:** Andrew L. Hufton

**Affiliations:** Editor-in-chief, *Patterns*, Cell Press

## Main text

In this month’s issue of *Patterns*, we take a special look at open-source software. Data science is a field that is defined more by a set of common tools and approaches than any other factor. These tools—statistical and databasing techniques, machine learning methods, etc.—are frequently embedded within and shared as open-source software. Indeed, open-source software could be viewed as the cord that binds together this otherwise diverse and multidisciplinary field.

On the cover this month is an artist’s rendering of a community garden, which we see as a metaphor for academic open-source software development. A single garden plot may serve a relatively small “user” group and may be tended by individuals with widely varying levels of gardening experience. At any point in time, some plots will be thriving, while others shelter weeds or lie fallow. Across the garden, tips, shovels, and labor are shared, maintaining the system through cooperation. Academic open-source software projects similarly must learn to succeed in this kind of ever-changing but consistently cooperative landscape.

To explore the role open-source software plays in data science from multiple angles, this special issue includes a selection of papers that present technical updates on major open-source software projects as well as papers that explore the more cultural and organizational aspects of open-source software development. Two additional papers consider the associated topics of open-source metadata standards development (Rokem et al.) and computational sustainability (Lee et al.).

The free and open-source software movements—which aim to promote software that can be widely used, modified, and shared—have been tightly connected with academic research since their inceptions, and many of the principles of sharing, cooperation, and freedom that define these movements have emerged, at least in part, from academic culture and its ideals. Today, open-source software is a key pillar of open science, a movement that aims to make science more accessible, reusable, and equitable (for more on these movements and their aims, see UNESCO’s 2023 recommendation on open science, Gonzalez-Barahona, 2021, *Computer*, and the Open Source Initiative’s history page).

While open-source software development often depends, at least in part, on contributions from non-professional programmers, research institutions are increasingly relying on professional “research software engineers” to supplement and guide more grassroots programming efforts. In this issue, Chue Hong et al. look back on the efforts made by the Software Sustainability Institute over the last 15 years to support the spread of this new profession and improve software used in research. Also in this special issue, we share an interview with two research software engineers working at the International Brain Laboratory to learn more about how they perceive open-source software development (Faulkner and Wells).

This special issue also serves as the formal launch of our new “resource” article type. This issue includes four resource papers that each present an update on a widely used open-source software project or tool. The resource paper format is designed to be flexible, allowing authors to provide technical information as well as more qualitative content on, for example, the organization, governance, and history of a project. New submissions are welcome under this format at any time. Submissions may describe new software tools, datasets, or other resources or may provide updates on existing, broadly used resources. For tips on selecting a license for an open-source software project, we recommend choosealicense.com as well as the more in-depth resources provided by the Open Source Initiative.

We hope that this special issue, and our new resource format, will help highlight the crucial role that open-source software development plays in academic research and will provide visibility to the often-overlooked tasks of maintaining, managing, and updating academic software.

